# A Fast and Highly Stable Aqueous Calcium‐Ion Battery for Sustainable Energy Storage

**DOI:** 10.1002/cssc.202401469

**Published:** 2024-11-14

**Authors:** Raphael L. Streng, Samuel Reiser, Sabrina Wager, Nykola Pommer, Aliaksandr S. Bandarenka

**Affiliations:** ^1^ Physics of Energy Conversion and Storage Physik-Department Technische Universität München James-Franck-Str. 1 85748 Garching Germany; ^2^ Catalysis Research Center TUM Ernst-Otto-Fischer-Straße 1 85748 Garching Germany

**Keywords:** Aqueous batteries, Calcium, Potassium, Polyimides

## Abstract

Aqueous alkali‐ion batteries are gaining traction as a low‐cost, sustainable alternative to conventional organic lithium‐ion batteries. However, the rapid degradation of commonly used electrode materials, such as Prussian Blue Analogs and carbonyl‐based organic compounds, continues to challenge the economic viability of these devices. While stability issues can be addressed by employing highly concentrated water‐in‐salt electrolytes, this approach often requires expensive and, in many cases, fluorinated salts. Here, we show that replacing monovalent K^+^ ions with divalent Ca^2+^ ions in the electrolyte significantly enhances the stability of both a copper hexacyanoferrate cathode and a polyimide anode. These findings have direct implications for developing an optimized aqueous Ca‐ion battery that demonstrates exceptional fast‐charging capabilities and ultra‐long cycle life and points toward applying Ca‐based batteries for large‐scale energy storage.

## Graphical abstract

Aqueous batteries provide a low‐cost, safer alternative to lithium‐ion batteries, but their viability is often limited by rapid electrode degradation. This study shows that replacing K^+^ with divalent Ca^2+^ ions in the electrolyte significantly boosts the stability of both copper hexacyanoferrate cathodes and polyimide anodes, enabling fast‐charging, long‐lasting aqueous Ca‐ion batteries made entirely from abundant, sustainable materials.

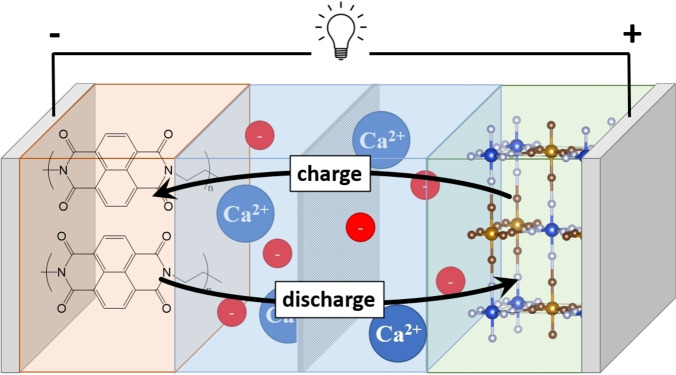

## 1 Introduction

Our societies’ ever‐rising energy demand, combined with the growing awareness of the negative implications of fossil fuel‐based energy production, has led to a transition toward renewable energy sources.[^1^, ^2^] However, the generation vs. consumption discrepancy of, e. g., solar or wind power still poses a significant challenge to energy supply safety and independence. Accordingly, large‐scale storage is crucial for the renewable energy transition.[^3^, ^4^, ^5^] There is a wide range of storage technologies, among which batteries are considered one of the most efficient and flexible.[^6^, ^7^] Due to their high energy density, Li‐ion batteries (LIBs) dominate the battery market for electric vehicles and portable electronics and are also a popular choice for stationary storage applications.^[8]^ However, the competition for scarce lithium resources and safety concerns related to the organic electrolyte have sparked a renewed interest in alternative systems employing safer and more available materials.[^9^, ^10^] Aqueous batteries based on highly abundant salts are promising candidates to replace LIB in stationary energy storage and certain public transport applications due to their inherent safety, sustainability, and fast‐charging capabilities..[^11^, ^12^, ^13^] At the same time, the main drawback of aqueous batteries is the relatively narrow electrochemical stability window, limiting the maximum cell voltage and, thus, the cell′s energy density.^[14]^ Therefore, for aqueous batteries to economically challenge LIBs, lowering production costs and improving cycling stability by mitigating degradation effects is paramount.^[15]^ However, most compounds that qualify as anode or cathode active materials suffer from rapid capacity fading.[^16^, ^17^, ^18^] As capacity loss is usually linked to active material dissolution in water, most stabilization approaches focus on reducing the amount of free water in the electrolyte by employing so‐called water‐in‐salt electrolytes.[^19^, ^20^, ^21^, ^22^] Herby, the high incorporation ratio of water molecules in the ion hydration shell mitigates hydrolysis of the anode and cathode. While being very successful, this strategy relies on using high amounts of expensive, often fluorinated salts, which contradicts the aim of reducing production costs.^[23]^ Thus, new approaches to improve the cycling stability of aqueous batteries should be investigated. In fact, stabilization measures such as electrolyte additives and protective coatings have been studied extensively.[^24^, ^25^, ^26^, ^27^, ^28^] Additionally, the effect of the electrolyte anions and alkali cations on electrode materials has been investigated in recent years.[^15^, ^29^, ^30^, ^31^] However, as most research focuses on aqueous batteries using either only monovalent ions such as Li^+^, Na^+^, and K^+^ or only divalent ions such as Mg^2+^ and Ca^2+^, the effect of exchanging monovalent cations for divalent cations is rarely discussed. This is where our research comes in, aiming to fill this gap and provide potential solutions for the challenges of aqueous batteries.

This study investigates a model battery consisting of an organic polyimide anode, a Prussian blue analog (PBA) copper hexacyanoferrate cathode, and an aqueous potassium or calcium chloride electrolyte. This combination is selected due to the promising properties of both electrode materials.[^32^, ^33^] The effect of the electrolyte cation on each electrode is tested separately before the cycling stability of the aqueous K‐ion and the aqueous Ca‐ion battery are compared. Finally, the Ca‐ion battery′s rate capability and energy density are assessed in more detail.

## 2 Materials and Methods

### 2.1 Material Synthesis

All chemicals for synthesis were purchased from commercial suppliers and used without further purification.

Poly(naphthalene four formyl ethylenediamine) (PNFE) was synthesized for our previous work and also used for this study without any further processing..^[34]^ In a typical procedure, 10 mmol (2.68 g) of 1,4,5,8‐naphthalene tetracarboxylic dianhydride (NTCDA) and 10 mmol (0.6 g) of ethylenediamine (EDA) were dissolved in 20 ml of degassed N‐methyl pyrrolidone (NMP) in a Schlenk flask. The mixture was heated to 215 °C in a sand bath and reacted under reflux overnight. The dark precipitate was filtered, washed with NMP and subsequently with water several times. Finally, it was dried under vacuum at 0.08 mbar stepwise at three different temperatures (room temperature, 120 °C, and 210 °C). The product was a grey‐brownish powder with a high yield of 92 % (2.7 g).

Elemental analysis of PNFE: Calcd. for an infinite polyimide (C_16_H_8_N_2_O_4_), %: C 65.76, H 2.76, N 9.59; calcd. for an octamer, %: C 65.10, H 3.03, N 10.52; found, %: C 65.13, H 2.91, N 10.32.

Copper hexacyanoferrate (CuHCF) was prepared by coprecipitation according to the method reported by Xu et al.^[35]^ 1 mmol of CuCl_2_ ⋅ 2H_2_O and 1 mmol of Na_4_P_2_O_7_ ⋅ 10 H_2_O were dissolved in 20 ml DI water to form solution A. Solution B was prepared by adding 1 mmol of Na_4_Fe(CN)_6_ ⋅ 10 H_2_O, 0.3 g of polyvinylpyrrolidone (PVP) and 0.58 g of NaCl into 50 ml DI water. Subsequently, solution A was dropwise added to solution B under vigorous stirring at room temperature. Subsequently, the mixture was left for aging for 5 days before the precipitate was collected by centrifugation and washed 3 times with water and ethanol, respectively. The product was dried overnight at 70 °C.

### 2.2 Electrode Preparation

PNFE and CuHCF electrodes were prepared using the slurry casting method. For the PNFE slurry 80 wt. % polyimide active material, 10 wt. % carbon black super C‐65 (MTI, USA) and 10 wt. % Nafion™ binder (5 wt. % in lower aliphatic alcohols and water, Sigma Aldrich, USA) was mixed and ground in a planetary ball mill. To form the anode slurry, water, and isopropyl alcohol in a 1 : 1 ratio were added as dispersing agents under vigorous stirring.

The CuHCF slurry was prepared using a similar procedure, mixing 80 wt % active material with 10 wt. % carbon black super C‐65 and 10 wt. % polyvinylidene fluoride (PVDF, MTI, USA) in a planetary ball mill. NMP was added under vigorous stirring to form the cathode slurry.

Both slurries were cast onto a pyrolytic graphite sheet current collector. The active material mass loading was approximately 4.0 mg cm^−2^ for the CuHCF cathode and 2.0 mg cm^−2^ for the PNFE anode.

### 2.3 Material Characterization

The morphology of the electrode films was investigated with a field emission scanning electron microscope (SEM, JSM‐7500F, JEOL). Fourier‐transformed infrared (FTIR, Bruker Vertex‐70) spectroscopy was used to characterize the chemical structure of PNFE, and powder x‐ray diffraction (XRD) was used to analyze the crystal structure of CuHCF. Thermogravimetric analysis (TGA) was performed under an argon atmosphere using a METTLER TOLEDO TGA/DSC 2 (STARe system). The analysis was conducted over a temperature range of 30 °C to 800 °C, with a heating rate of 10 °C per minute. X‐ray photoelectron spectroscopy (XPS) measurements of the PNFE electrode were obtained using a SPECS system, equipped with a non‐monochromatized Al Kα source (1486.7 eV), an SPECS XR50 X‐ray source, a SPECS PHOIBOS 150 hemispherical analyzer, and an SPECS spectrometer. XPS spectra were analyzed and fitted using Casa XPS software (Version 2.3.24PR1.0), with binding energies adjusted by setting the C–C peak in the C 1s spectrum to 284.8 eV.

### 2.4 Electrochemical Measurements

Single‐electrode measurements were conducted in a three‐electrode setup inside a closed glass cell with constant argon flow to ensure and maintain an inert atmosphere. The electrolyte and the glass cell were purged with argon for at least 10 minutes before the electrolyte was run into the cell. A platinum wire (MaTeck, Germany) was used as the counter electrode. The reference electrode was an Ag/AgCl electrode (SSC, 3 M KCl, SI Analytics) connected to the electrolyte via a Luggin capillary. 3 M and saturated KCl and CaCl_2_ were employed as electrolytes.

Cyclic voltammetry (CV) and galvanostatic cycling with potential limitations (GCPL) were conducted using a BioLogic VSP‐300 potentiostat. Cyclic voltammograms (CVs) were recorded at different scan rates ranging from 1 mV s^−1^ to 50 mV s^−1^ at potentials from −1.0 V to 0.0 V and from 0.3 V to 1.1 V (vs. SSC) for anode and cathode, respectively. Galvanostatic (dis−)charging was carried out at C‐rates between 1 C and 300 C within the same potential ranges. The cycling stability in different electrolytes was tested by galvanostatically charging and discharging the electrodes for 100 cycles at 10 C (cathode) and 1000 cycles at 100 C (anode).

Potentiostatic electrochemical impedance spectroscopy (PEIS) was carried out at −500 mV vs. SSC for the anode and at 800 mV vs. SSC for the cathode. The potential was held for 10 minutes before recording the spectrum to ensure equilibrium conditions. The AC amplitude was set to 10 mV, and a frequency range from 1.00 MHz to 250 MHz was used. Before fitting the data, the validity was verified by Kramers‐Kronig analysis.

### 2.5 Full‐Cell Assembly and Measurements

Due to the corrosive interaction of chlorides with most stainless steel alloys, a 3D‐printed plastic cell with pyrolytic graphite foil as contacts was used for the full cell. The aqueous K‐ion and Ca‐ion batteries were assembled under a nitrogen atmosphere inside a glovebox to avoid dissolved oxygen in the electrolyte. A commercial BS 17–25 separator (Mativ Inc., France) for alkaline batteries soaked in the respective electrolyte was placed between the electrodes. The closed cell was fully sealed by rubber o‐rings.

CV and GCPL measurements were also conducted with a Biologic VSP‐300 potentiostat for the full cell. CVs were recorded at scan rates from 1 mV s^−1^ to 50 mV^−1^ between cell voltages of 0.6 V, and 1.7 V. GCPL was conducted at C‐rates between 300 C and 1 C in the same voltage range.

## 3 Results and Discussion

**Figure 1:**
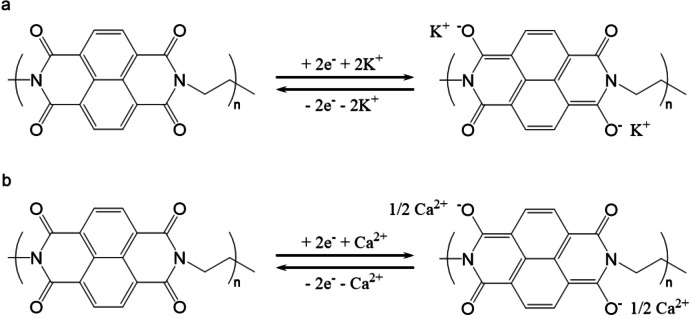
Reduction and oxidation mechanism of PNFE. Redox mechanism of PNFE in the presence of (a) K‐ions and (b) Ca‐ions.

Organic electrode materials have attracted increased attention due to their exceptional sustainability, tunability, and compatibility with aqueous electrolytes. Several high‐performance materials have been developed in recent years, including conductive polymers, carbonyl compounds, radicals, and organic frameworks.[^32^, ^36^, ^37^, ^38^, ^39^] Among them, polyimides are regarded as one of the most promising anode materials for aqueous batteries and have been used in various aqueous Li‐ion, Na‐ion, and K‐ion batteries.[^40^, ^41^] PNFE was synthesized in a one‐step dehydration condensation reaction between NTCDA and EDA in NMP in our previous work^[34]^ and used as an anode in this study. FTIR, TGA, and XPS analysis confirming the successful synthesis of PNFE is shown in Figure S1 (replotted from^[34]^). The observed FTIR pattern is in good accordance with the expected structure of PNFE. In particular, the bands at 1626 cm^−1^ and 1580 cm^−1^ can be attributed to stretching vibrations of the CC bonds in the cyclic alkenes. Furthermore, various vibrations of aromatic and non‐aromatic C−N bonds (1348 cm^−1^ to 1094 cm^−1^) and stretching vibrations of the C=O bonds in the electrochemically active carbonyl groups (1701 cm^−1^ and 1661 cm^−1^) can be observed. The remaining spectral bands correspond to vibrations of C−H bonds in the polymer. In the XPS survey scan, characteristic peaks for fluorine, oxygen, carbon, and nitrogen can be observed corresponding to the constituents of the Nafion binder and the active material. The fitted C 1s spectrum shows sharp peaks for the contributions from C−C, C−N, C=O, and CF_2_ bonds and weaker peaks that can be attributed to C=C, N−C=O, and CF_3_ bonds. The structural stability of PNFE upon ball milling was confirmed by a subsequent FTIR measurement (Figure S3). The reduction mechanism of PNFE in the presence of monovalent ions such as K^+^ is shown in Figure 1(a). It involves a two‐step enolization of two opposite carbonyl groups during which the charge within the aromatic rings is redistributed.^[34]^ The remaining carbonyl groups can generally be reduced as well. However, this reduction requires potentials below the hydrogen evolution reaction (HER) threshold, making it unfeasible in aqueous batteries.

In addition to monovalent ions, polyimides have also been shown to host divalent cations such as Mg^2+^ and Ca^2+^.[^42^, ^43^] The associated mechanism is illustrated in Figure 1(b). In this process, carbonyl groups from two neighboring monomers participate in hosting a single divalent cation. Consequently, the total transferred charge is equivalent to that of monovalent ion insertion.

**Figure 2:**
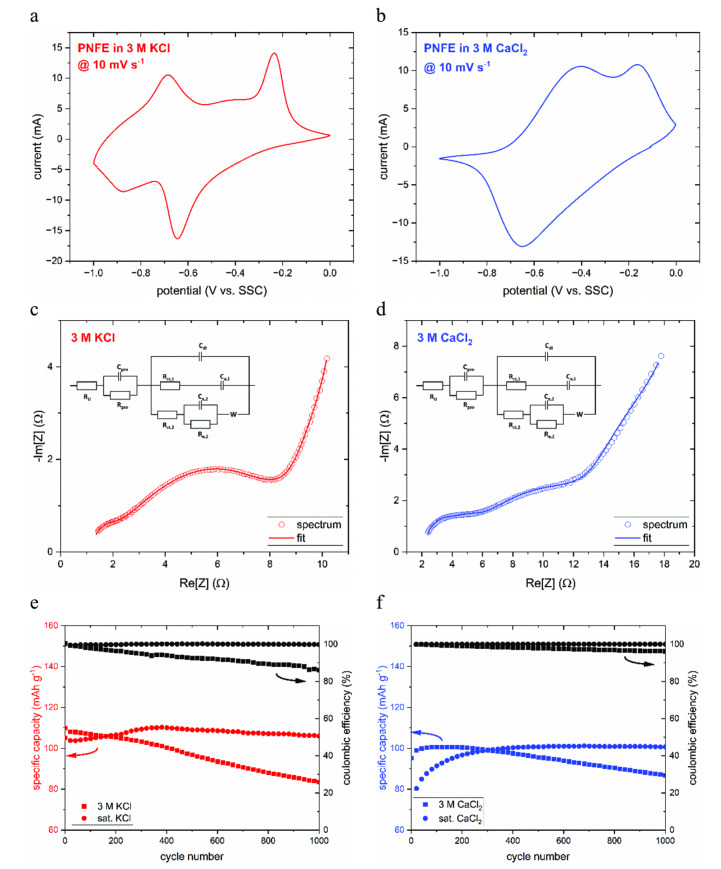
CV and stability measurements of PNFE electrodes in KCl and CaCl_2_ electrolytes. (a) CV of a PNFE electrode in 3 M KCl at 10 mV s^−1^. (b) CV of a PNFE electrode in 3 M CaCl_2_ at 10 mV s^−1^. (c) Fitted impedance spectrum of a PNFE electrode in 3 M KCl at −500 mV vs. SSC. (d) Fitted impedance spectrum of a PNFE electrode in 3 M CaCl_2_ at −500 mV vs. SSC. (e) Cycling stability of PNFE in 3 M and saturated KCl recorded over 1000 cycles at 100 C. (f) Cycling stability of PNFE in 3 M and saturated CaCl_2_.

To compare the electrochemical behavior of PNFE in aqueous electrolytes containing either monovalent or divalent ions, CVs were recorded at 10 mV s^−1^ in 3 M KCl and CaCl_2_ solutions. The resulting voltammograms are shown in Figure 2(a) and (b**)**. In the presence of K‐ions, two reduction peaks (−0.65 V, −0.87 V vs. SSC) and two oxidation peaks (−0.23 V, −0.68 V vs. SSC) appear, which is in good accordance with the previously introduced two‐step redox mechanism.

In CaCl_2_, the shape of the CV, as well as the position of the peaks, differs significantly. The reduction peaks overlap at −0.65 V vs. SSC, and the oxidation peaks are located at −0.40 and −0.15 V vs. SSC, respectively. Thus, PNFE exhibits a slightly higher half‐charge potential in the presence of Ca‐ions, as the higher charge density of the divalent ion facilitates the enolization process. To further analyze the influence of the cation on the reduction mechanism of PNFE, potentiostatic electrochemical impedance spectroscopy (PEIS) measurements were carried out in both electrolytes at −500 mV vs. SSC. Figures 2(c) and (d) show the fitted impedance spectra of the anode in 3 M KCl and 3 M CaCl_2_, respectively, as well as the equivalent circuit used to fit the data. This impedance model was demonstrated to accurately describe the charge storage mechanism of PNFE in our previous work and can be used to fit the shown data accurately with a root mean square deviation of less than 1 %.^[34]^ It is derived from the so‐called Randles circuit, including the uncompensated electrolyte resistance *R_U_*, the double layer capacity *C_dl_*, the charge transfer resistance *R_ct_*, and a semi‐infinite Warburg element to account for diffusion. The presence of the adsorption capacitance and resistance (*C*_*a,2*_ and *R*_*a,2*_) in both electrolytes indicates that the electrode material goes through a two‐step reduction process regardless of the cation species supporting the reduction mechanism shown in Figure 1. In addition, a secondary single‐electron reduction pathway (*R*_*ct,1*_ and *C*_*a,1*_) can be observed in both cases, corresponding to the reduction of only one carbonyl group per polymer unit with subsequent stabilization of the radical anion due to polymer‐polymer interactions. However, in 3 M KCl and 3 M CaCl_2_, *R*_*ct,1*_ is ~15 times and ~10 times higher than *R*_*ct,2*_, respectively, indicating that this pathway is significantly suppressed compared to the main two‐step reduction. The parameters *C_geo_* and *R_geo_* account for the contribution of the film on the graphite sheet. For a more detailed discussion of the impedance model, the reader is referred to our previous work.^[34]^ Based on the PEIS analysis, two reduction steps are detected in both electrolytes. Thus, although only one cathodic peak can be observed in the CV in the CaCl_2_ electrolyte, it actually represents the merging of the two peaks observed in the KCl electrolyte. The overlap of the potentials of the two reduction steps can be explained by the interplay between multiple monomers involved in hosting a single Ca‐ion. As multiple neighboring polymer units take part in the insertion of divalent ions, the two reduction steps can no longer be distinguished as two clearly separated peaks. The two naphthalene diimide groups participating in the adsorption of a single Ca^2+^ cation may be in different oxidation states, causing the peaks to merge. When a Ca‐ion is attracted by a reduced carbonyl group, its electron‐attracting nature will facilitate the reduction of adjacent carbonyl groups. Therefore, the reduction of the different polymer units becomes more conjugated, affecting the CV shape. In addition, repulsive interactions between the redox centers lead to further peak broadening. These findings demonstrate that the electrochemical behavior of PNFE can be tailored by using different cations affecting electrode potential and reversibility.

Furthermore, the influence of the electrolyte composition on the cycling stability of PNFE was investigated by repeatedly charging and discharging the electrode for 1000 cycles in both electrolytes. A high cycling rate of 100 C (36 s per (dis−)charge cycle) was chosen as PNFE exhibits an exceptionally high capacity retention of ~70 % at this rate. Figures 2(e) and (f) show the specific discharge capacity of the anode in KCl and CaCl_2_, respectively. In 3 M KCl, the initial discharge capacity is ~110 mAh g^−1^ but decays significantly, with only ~75 % of the capacity remaining after 1000 cycles.

Due to the stronger interaction and, therefore, slower diffusion of Ca‐ions, the initial capacity at 100 C is slightly lower in 3 M CaCl_2_. However, the capacity retention is improved (~87 % after 100 cycles), which can be attributed to the higher concentration of water‐binding anions and the stronger interaction between the divalent Ca‐ions and the water dipoles. Harmful interactions with the anode become less likely as the water molecules are more strongly bound to the cations.

As mentioned previously, electrode degradation is commonly associated with active material dissolution into the aqueous electrolyte. It can be mitigated by increasing the salt concentration and limiting the amount of free water. Therefore, the cycling stability was also evaluated in saturated KCl (4.7 mol kg^−1^) and saturated CaCl_2_ (6.7 mol kg^−1^). PNFE exhibits exceptional cycling stability in both solutions without significant degradation after 100 cycles. In CaCl_2,_ a precycling phase with increasing capacity can be observed, which is most likely linked to slow electrolyte penetration into the pores of the slurry due to the higher viscosity of the saturated solution. It becomes clear that whereas sufficient capacity retention in PNFE anodes can be achieved in a saturated KCl electrolyte, replacing K^+^ with Ca^2+^ generally impedes degradation effects. In addition to the reasons listed above, electrolyte viscosity can play a role, as the transport of dissolution products away from the electrode‐electrolyte interface is needed for further dissolution.

**Figure 3:**
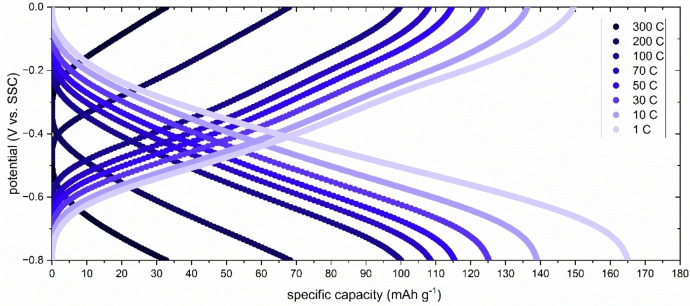
Rate performance of PNFE in saturated CaCl_2_. Galvanostatic (dis−) charge curves of a PNFE electrode measured at C‐rates ranging from 300 C (12 s) to 1 C (1 h) in a saturated CaCl_2_ aqueous electrolyte.

Figure 3 shows the rate performance of PNFE in saturated CaCl_2_. Galvanostatic cycling was carried out at C‐rates ranging from 1 C to high rates of up to 300 C.

At 1 C, a maximum discharge capacity of ca 150 mAh g^−1^ with a Coulombic efficiency of ~90 % can be observed. Slight losses can be explained by the parasitic HER, which is more pronounced in the large electrolyte volume of the three‐electrode setup than in real‐life cells that only contain minimal amounts of water. As a pyrolytic graphite sheet is used as the current collector, it should be noted that graphite can store Ca‐ions with considerable capacities in non‐aqueous electrolytes. However, at 0.5–1.0 V vs. Ca^2+^/Ca the potential for calcium intercalation into graphite is far below the potentials used in the measurements above.^[44]^ Accordingly, preliminary CV measurements of the current collector in the aqueous CaCl_2_ electrolytes showed no capacity contributions.

At increasing C‐rates, the capacity retention remains high, with a discharge capacity of 135 mAh g^−1^ at 10 C and over 100 mAh g^−1^ at 100 C. This agrees with previous studies showing that PNFE exhibits pseudocapacitive behavior with low diffusion limitations.[^34^, ^43^] Even in the more viscous saturated CaCl_2_ electrolyte, the anode demonstrates exceptional fast charging capability, allowing supercapacitor‐like applications.

CuHCF, which was selected as a suitable cathode material for the aqueous battery studied in this work, was synthesized by a modified coprecipitation, as reported by Xu et al.^[35]^ They found that the addition of the chelating agents Na_4_P_2_O_7_ and PVP during the coprecipitation improved the crystallinity of the CuHCF particles. Furthermore, they observed a change in crystal structure from cubic to monoclinic when the precipitation occurs in the presence of concentrated NaCl. Highly crystalline nanosheets are formed in this configuration, as shown in the SEM pictures in Figure S2 (b) and (c). The successful synthesis of CuHCF nanosheets was further confirmed by powder XRD (Figure S2a). To assess the stability of CuHCF upon ball milling, the XRD spectrum was again recorded after this processing step (Figure S3).

**Figure 4:**
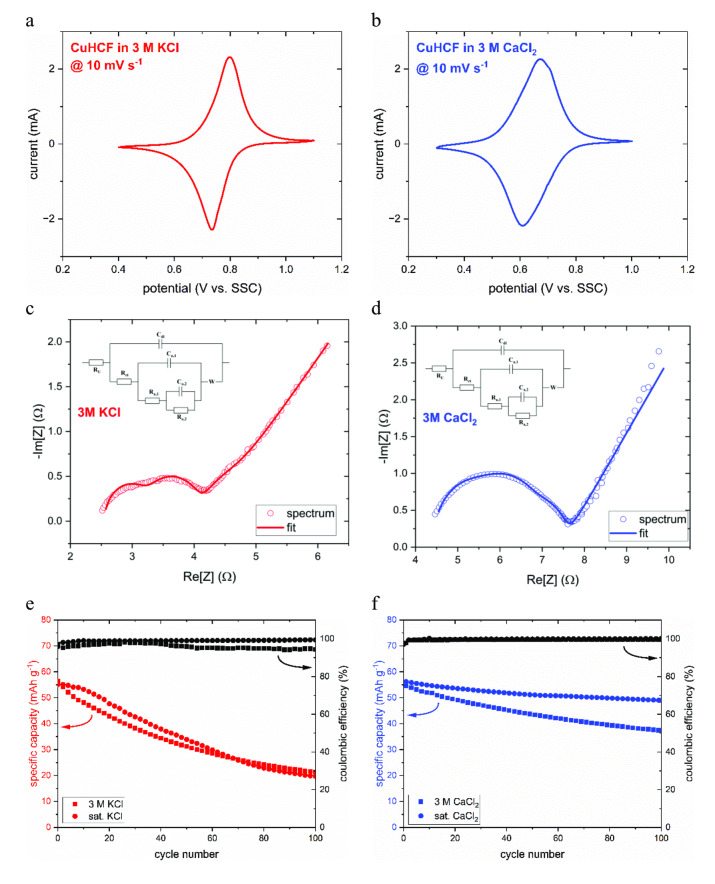
CV and stability measurements of CuHCF electrodes in KCl and CaCl_2_ electrolytes. (a) CV of a CuHCF electrode in 3 M KCl at 10 mV s^−1^. (b) CV of a CuHCF electrode in 3 M CaCl_2_ at 10 mV s^−1^. (c) Fitted impedance spectrum of a CuHCF electrode in 3 M KCl at 800 mV vs. SSC. (d) Fitted impedance spectrum of a CuHCF electrode in 3 M CaCl_2_ at 700 mV vs. SSC. (e) Cycling stability of CuHCF in 3 M and saturated KCl recorded over 1000 cycles at 100 C. (f) Cycling stability of CuHCF in 3 M and saturated CaCl_2_.

The electrochemical behavior of CuHCF was investigated in 3 M KCl and 3 M CaCl_2_, similar to the previous investigations on the PNFE anode. Figure 4(a) and (b) show the CVs of the cathode recorded at a scan rate of 10 mV s^−1^. K‐ions’ intercalation manifests itself by a reduction peak located at 0.74 V vs. SSC, with the corresponding oxidation peak at 0.80 V vs. SSC. In the CaCl_2_ electrolyte, the peaks are shifted towards lower potentials at ca 0.61 V and ca 0.68 V vs. SSC, respectively. In 2018, Bors et al. reported a distinct relationship between the hydration energy of alkali metal ions and the intercalation potential of various PBAs. Generally, more negative hydration energy (e. g., Li^+^: −520 kJ mol^−1^) leads to lower redox potentials if compared to more positive hydration energy (e. g., Cs^+^: −276 kJ mol^−1^). However, to our knowledge, no studies have reported a similar relation, including both monovalent and divalent cations. While the hydration energy of Ca^2+^ at −1577 kJ mol^−1^ is indeed lower than that of K^+^ at −322 kJ mol^−1^, the potential difference is relatively low, considering the large hydration energy gap.

The charge transfer mechanism of the cathode in both electrolytes was further investigated by impedance spectroscopy. To account for the redox potential shift depending on the intercalating species, the potential was set to 800 mV vs. SSC for KCl and 700 mV vs. SSC for CaCl_2_. Figures 4(c) and (d) show the fitted spectra and the used equivalent circuit. The model cation intercalation into PBAs in aqueous media was adapted from Yun et al. and represents a three‐step mechanism, including an electroactive step, a specific adsorption step, and a non‐electroactive step.^[45]^ The electroactive step of the intercalation into PBAs corresponds to the fast‐proceeding oxidation of Fe^II^. Subsequently, anions specifically adsorb on the electrode surface to compensate for the temporary excess positive charge due to slow cation de‐intercalation. In the last step, the cations and anions leave the surface. In the case of thicker slurry electrodes, the model is expanded by a semi‐infinite Warburg element to account for solid‐state and pore diffusion. In the 3 M CaCl_2_ electrolyte, the charge transfer resistance (R_ct_) and the resistances representing the adsorption (R_a,1_) and the non‐electroactive step (R_a,2_) are about 50 % higher than in the 3 M KCl solution, whereas the Warburg coefficient is not influenced by the cation species. In both cases, the resistances are in the range of 0.5–2.0 Ω, and the Warburg coefficients lie at 4.5 Ω s^−1/2^ (KCl) and 4.3 Ω s^−1/2^ (CaCl_2_) for an electrode with a surface area of 0.5 cm^2^ and a mass loading of 3.9 mg cm^2^. Generally, the diffusion of multivalent ions such as Mg^2+^ or Al^3+^ is significantly more sluggish than for monovalent ions due to their high charge density and, therefore, strong interaction with the host lattice. However, the relatively large ionic radius and lower charge density of Ca‐ions likely facilitate ion diffusion leading to a similar diffusion coefficient as for K‐ions.^[42]^ However, the deintercalation and desorption are more strongly impeded in the case of Ca^2+^, which is likely due to the temporary and locally higher charge density at the electrode surface during the oxidation process in the presence of multivalent ions. Thus, although the voltage polarization in the presence of K‐ions is expected to be slightly lower, the rate capability is generally high for both systems. Particularly at high currents, where diffusion becomes a main limiting factor, the performance will not be as strongly affected by the cation species.

To evaluate the cation effect on the stability of CuHCF, the electrode was cycled at 10 C for 100 cycles in KCl and CaCl_2_ electrolytes. Due to the more diffusion‐limited processes in CuHCF compared to PNFE, a lower (dis−)charging rate was chosen. Figures 4(e) and (f) show the resulting specific capacity per cycle. Both in 3 M and saturated KCl, the cathode degrades rapidly, with only ~40 % of the initial capacity remaining after 100 cycles. This strong capacity degradation can be explained by several mechanisms that have been proposed in previous studies. Generally, lattice defects caused by Fe(CN)_6_ vacancies generated during material synthesis have been shown to result in structural instability and capacity fading.[^33^, ^46^, ^47^] Additionally, multiple groups have investigated the detrimental effect of interstitial water on PBA performance and cycling stability. For example, Song et al. and Kim et al. have reported significant stability improvements induced by the removal of interstitial water during material synthesis for MnHCF and CuHCF, respectively.[^48^, ^49^] PBAs are also known to degrade rapidly under alkaline conditions and suffer from capacity decay due to the attack on Fe‐sites by OH^−^.[^50^, ^51^, ^52^]

In 3 M CaCl_2,_ however, the capacity retention improves significantly to ~70 %, which can be attributed to two main effects: First, the electrolyte pH decreases due to the reactivity of Ca‐ions with OH‐ions. As mentioned above, the detrimental impact of OH^−^‐adsorption on the stability of PBAs has been demonstrated previously by multiple studies. Thus, the acidity of the CaCl_2_ electrolyte can be expected to mitigate dissolution effects by minimizing the presence of harmful OH^−^ species. Although small, a certain concentration of OH^−^ is present in all aqueous electrolytes. Hence, particularly at electrodes that operate close to the oxygen evolution potential, the specific adsorption of OH‐ions can lead to local pH changes and promote degradation. Furthermore, Ojwang et al. have demonstrated a two‐step degradation mechanism for FeHCF that involves a redox reaction between the PBA and water in the reversible first step, inducing locally alkaline conditions. Subsequently, an irreversible structural decomposition under basic conditions occurs in the second step.^[53]^ This effect may be mitigated in Ca‐based electrolytes as OH‐ions are captured by the strongly interacting Ca‐ions. Second, as discussed above, the higher Cl^−^ concentration and the stronger hydration of Ca^2+^ decrease the amount of free water and increase viscosity, further impeding dissolution. It has been shown that the structural decomposition of PBAs can be reversed in the presence of the respective transition metal cation.^[54]^ Thus, the viscous CaCl_2_ electrolyte may improve the cycling stability by preventing the transport of dissolution products away from the electrode. This effect can be amplified using saturated CaCl_2_, leading to a capacity retention of ~90 %. Although cathode degradation is still observable, it is significantly suppressed and should be even less pronounced within a battery cell containing only minimal amounts of electrolyte.

**Figure 5:**
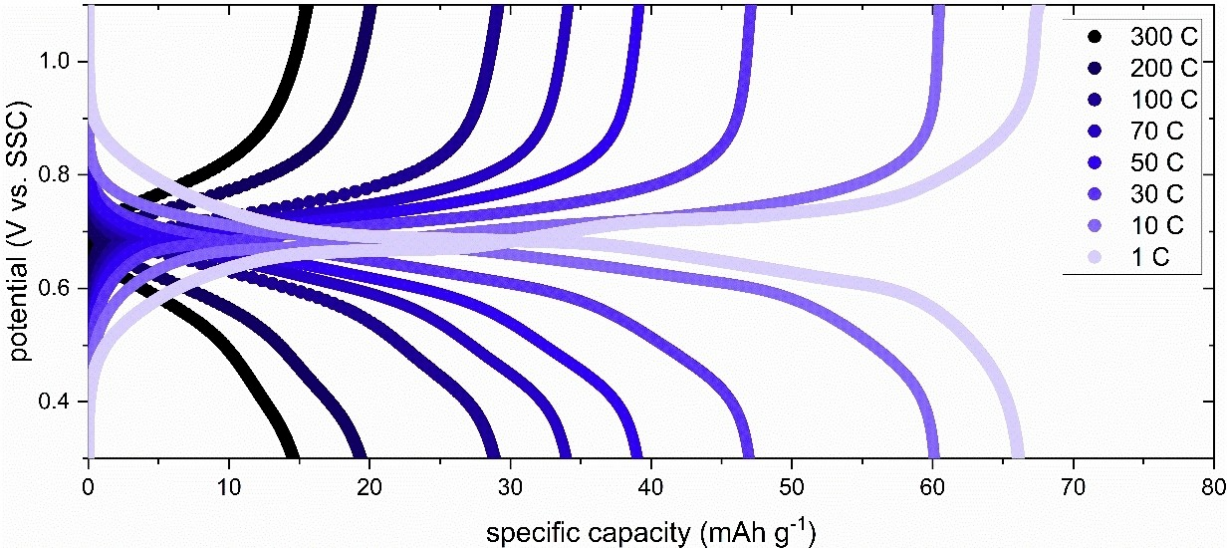
Rate performance of CuHCF in saturated CaCl_2_. Galvanostatic (dis−) charge curves of a CuHCF electrode measured at C‐rates ranging from 300 C (12 s) to 1 C (1 h) in a saturated CaCl_2_ aqueous electrolyte.

Figure 5 shows the galvanostatic charge and discharge curves of the CuHCF cathode recorded at C‐rates ranging from 1 C to 300 C. The electrode displays a maximum discharge capacity of ca 65 mAh g^−1^ with a Coulombic efficiency of nearly 100 % at 1 C. With capacities of ca 60 mAh g^−1^ and ca 34 mAh g^−1^ at 10 C and 100 C, respectively, the fast‐charging capabilities of CuHCF are considerably inferior to those of PNFE at similar mass loadings. Thus, the rate capability of the full cell can be influenced by controlling the mass balancing between the anode and the cathode. An oversized cathode will increase the specific capacity at high rates, whereas balanced anode and cathode capacities are favorable for slow‐charging applications.

**Figure 6:**
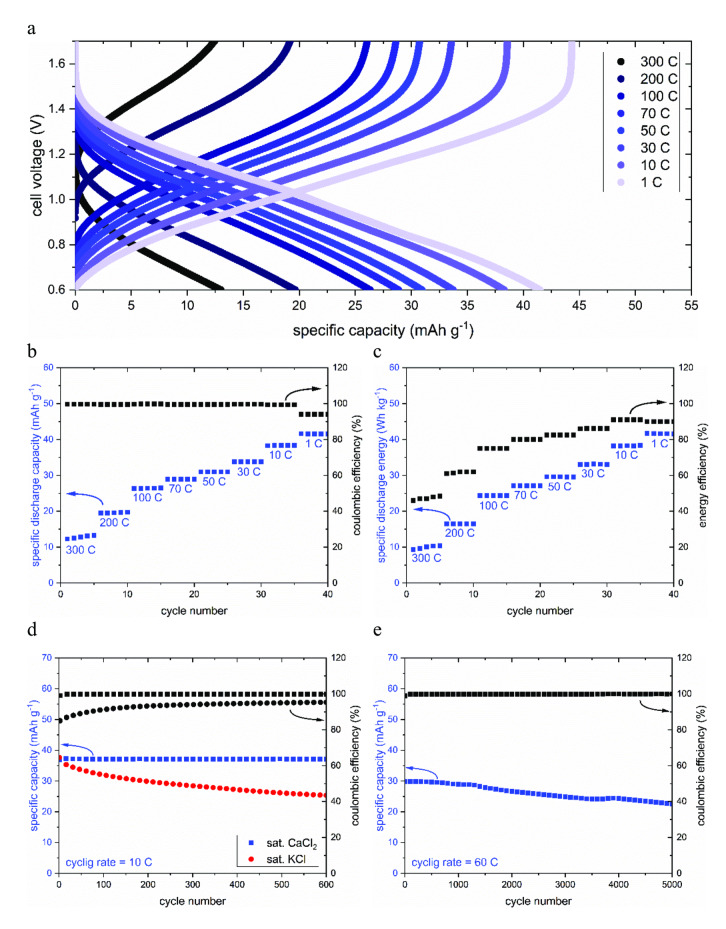
Performance of the CuHCF/CaCl_2_/PNFE full cell. (a) Galvanostatic (dis−) charge curves. (b) Specific discharge capacity and Coulombic efficiency per cycle at different cycling rates. (c) Specific energy and energy efficiency per cycle. (d) Cycling stability measured over 600 cycles at 10 C. (e) Cycling stability measured over 5000 cycles at 60 C. The specific capacity and energy are normalized to anode and cathode active material.

For this study, a mass‐balanced cell was assembled with an anode‐to‐cathode active material ratio of 1 : 2. Based on the previous findings regarding electrode stability, saturated CaCl_2_ was used as the electrolyte. The galvanostatic charge and discharge curves of this battery are shown in Figure 6(a). 5 cycles were recorded at each C‐rate to ensure reversibility and monitor any degradation at different C‐rates. At 1 C, the voltage profile shows a sloping plateau between 0.6 V and 1.5 V. In the voltage window from 0.6 V to 1.7 V, a reversible specific capacity of ca 41 mAh g^−1^ is observed, which amounts to ~90 % of the expected value, taking into account a cathode capacity of 65 mAh g^−1^ and an anode capacity of 150 mAh g^−1^. The battery exhibits exceptional fast charging capabilities, showing capacity retention of ca 88 % at 10 C, ca 66 % at 100 C, and ca 46 % at 200 C, respectively. At C‐rates above 10 C, the Coulombic efficiency approaches 100 %, indicating good reversibility, as shown in Figure 6(b).

The specific discharge energy per cycle, which is calculated by integrating the discharge curves, is shown in Figure 6(c). Due to increasing voltage polarization at higher currents, the specific energy exhibits a greater C‐rate dependence than the specific capacity. It drops from 41 Wh kg^−1^ at 1 C to 35 Wh kg^−1^ at 10 C (normalized to anode and cathode active material), indicating that the battery can be almost fully charged within minutes, nevertheless. Even at 100 C, more than 60 % of the maximum energy density is still reached, making this battery a viable choice for supercapacitor‐like use cases, e. g., in mobile applications that require lower ranges but short charging times. This conclusion is supported by evaluating the power density of the cell, which is calculated by multiplying the specific current with the average discharge voltage at each C‐rate. The specific current ranges from 41 mA g^−1^ (1 C) to 12.3 A g^−1^ (300 C), while the average discharge voltage lies between 1.00 V (1 C) and 0.81 V (300 C). This yields power densities between ~40 W kg^−1^ at 1 C and up to ~10 kW kg^−1^ at 300 C, which are, to our knowledge, among the highest reported for aqueous batteries so far.

**Figure 7:**
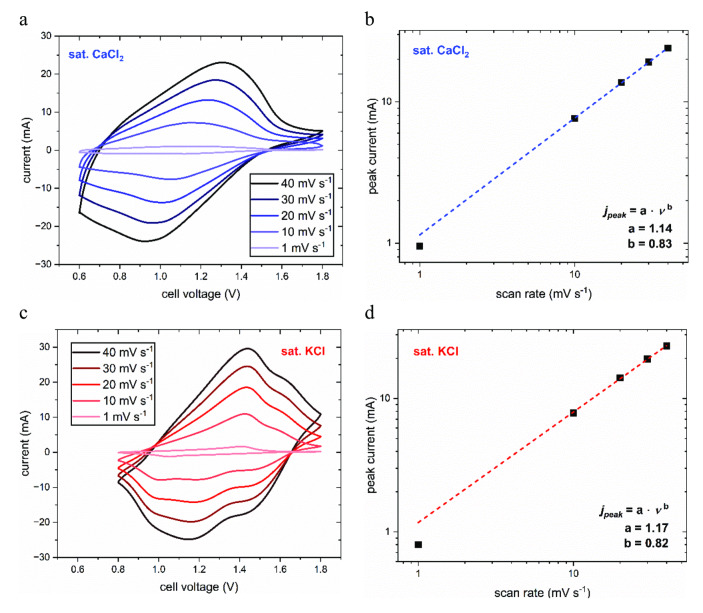
CVs of the sat. CaCl_2_ and the sat. KCl full cell. (a) CVs of the Ca‐ion battery at different scan rates. (b) Dependence of the reduction peak current on the scan rate for the Ca‐ion battery. (c) CVs of the K‐ion battery. (d) Dependence of the peak current on the scan rate for the K‐ion battery.

To confirm the beneficial influence of the Ca‐ion electrolyte on the cycling stability of the aqueous battery, cells containing either a saturated KCl or a saturated CaCl_2_ electrolyte were continuously charged and discharged at an intermediate rate of 10 C for 600 cycles. While the aqueous K‐ion battery only retains ~65 % of its initial capacity, the aqueous Ca‐ion battery maintains its capacity throughout the measurement. Furthermore, it displays a higher Coulombic efficiency due to suppressed water splitting side reactions in the concentrated CaCl_2_ electrolyte (Figure 6(d)). The stability under long‐term fast cycling was investigated by cycling the Ca‐ion battery for 5000 cycles at 60 C. Even under these high‐stress conditions, it maintained nearly 80 % of its initial capacity at the end of the measurement, indicating excellent high‐rate stability (Figure 6(e)). To further analyze the influence of the cation species on the characteristics of the full cell and the diffusion kinetics in the electrodes, CVs of the K‐ion battery and the Ca‐ion battery were recorded at different scan rates ranging from 1 mV s^−1^ to 40 mV s^−1^. The resulting voltammograms can be seen in Figure 7(a) and (c), respectively.

As expected from the single‐electrode measurements, the reduction and oxidation peaks are found at slightly higher voltages for the K‐ion battery since the electrode potential difference is larger. Although the energy density could, therefore, be maximized by using K^+^ ions, the vast stability improvements with Ca^2+^ ions outweigh this drawback. For both batteries, the peak‐to‐peak separation increases at larger scan rates, which can be mostly attributed to the uncompensated internal resistance of the cells. However, the low peak‐to‐peak separation at 1 mV s^−1^ indicates excellent reversibility of the charging processes, which is reflected in the aforementioned high energy efficiency at low C‐rates. Relating the peak current *j_peak_* to the scan rate *ν*, furthermore, reveals information on the diffusion kinetics in the electrodes:

(1) $$j_{peak}=a\cdot \nu^{b}$$

For fully surface‐limited processes like adsorption, the value of parameter *b* lies at 1.0, whereas it is 0.5 for fully mass‐transport‐limited processes. Figures 7(b) and (e**)** show the peak current of the main cathodic peak of the CV for both cells plotted against the scan rate with a fit according to equation (1). Interestingly, the value of parameter *b* does not change significantly when monovalent K‐ions are replaced with divalent Ca‐ions in the aqueous battery. Lying at ~0.8, it implies that the charging and discharging processes are partially mass‐transport‐limited for both cells. Since the charge storage mechanism of PNFE is quasi‐capacitive and, thus, mostly surface‐limited,[^34^, ^43^] the mass transport limitations mainly stem from solid‐state‐diffusion kinetics at the CuHCF cathode. As multivalent ions typically have higher charge densities and, thus, higher polarization strengths than monovalent ions, the solid‐state diffusion becomes more sluggish due to the stronger interactions between the cation and the host lattice. However, the relatively large ionic radius of Ca^2+^ leads to a significantly lower charge density than for other multivalent ions used in batteries, such as Al^3+^ and Mg^2+^.^[42]^ Consequently, the use of Ca‐ions instead of K‐ions does not impair the fast diffusion kinetics as severely, maintaining the high rate capability of the battery, which confirms the observations from the impedance measurements for the cathode.

The electrochemical performance of the full cell presented in this work is compared to other state‐of‐the‐art aqueous Ca‐ion batteries in Figure S4, demonstrating the superior power and energy density of this battery. Whereas an aqueous sulfur/Ca_0.4_MnO_2_ battery reported by Tang et al. in 2021 showed impressive energy densities of up to ~100 Wh kg^−1^, the slow kinetics of this system severely limit its power density.^[55]^ Energy densities similar to our work were achieved by Gheytani et al., Zhou et al., Zhang et al., and Wang et al.[^36^, ^42^, ^56^, ^57^] In addition, several other groups have developed high‐performance aqueous Ca‐ion batteries which are not included in the comparison due to missing data on energy and power density.[^58^, ^59^] However, power densities exceeding 10 kW kg^−1^ have not been reported for aqueous Ca‐ion batteries to the best of our knowledge.

## 4 Conclusions

In conclusion, this work demonstrates that the cation choice affects the stability of electrode materials in aqueous batteries. Besides the influence on the redox potential and behavior, the organic polyimide anode and the copper hexacyanoferrate cathode demonstrated an elongated cycle life when using a Ca‐ion‐based instead of a K‐ion‐based electrolyte. While the degradation of the anode could already be mitigated by saturating the aqueous KCl electrolyte, such an effect could not be observed for the cathode. However, the stability of the cathode was largely improved in a 3 M CaCl_2_ electrolyte and, more so, in a saturated CaCl_2_ electrolyte.

Similar findings could be made for the full cell employing both previously investigated electrodes. A rapid capacity decay was observed for the aqueous K‐ion battery, whereas the Ca‐ion battery showed no significant degradation after 600 cycles at 10 C. Consequently, the performance of the latter cell was further analyzed, demonstrating an energy density of ~41 Wh kg^−1^ at 1 C and a maximum power density of ~10 kW kg^−1^ at 300 C. Due to these exceptional fast‐charging properties, the stability was also evaluated at a fast cycling rate of 60 C. Under these conditions, the aqueous Ca‐ion battery maintained a high capacity retention of nearly 80 % after 5000 cycles.

Aside from the significant findings regarding the influence of the cation choice on the performance of aqueous batteries, this work helps to pave the way toward the practical implementation of aqueous batteries in large‐scale energy storage. The battery proposed in this study uses only low‐cost and highly available materials and promises to further advance the development of sustainable battery systems.

## Author Contributions

R. Streng and A. Bandarenka proposed the concept. R. Streng, S. Reiser, S. Wager, and N. Pommer performed the experiments. R. Streng and A. Bandarenka co‐wrote the manuscript. All authors participated in data analysis and manuscript discussion.

## Conflict of Interests

The authors declare no competing financial interests.

5

## Supporting information

As a service to our authors and readers, this journal provides supporting information supplied by the authors. Such materials are peer reviewed and may be re‐organized for online delivery, but are not copy‐edited or typeset. Technical support issues arising from supporting information (other than missing files) should be addressed to the authors.

Supporting Information

## Data Availability

The data that support the findings of this study are available from the corresponding author upon reasonable request.
