# Supplementary material for: A Fast and Highly Stable Aqueous Calcium‐Ion Battery for Sustainable Energy Storage
Source: ChemSusChem. 2024 Nov 14;18(6):e202401469. doi: 10.1002/cssc.202401469 (PMC11912106; doi:10.1002/cssc.202401469)
Supplement: Supplementary file 1 — Supporting Information [file CSSC-18-e202401469-s001.pdf]

ChemSusChem

Supporting Information

A Fast and Highly Stable Aqueous Calcium-Ion Battery for Sustainable Energy Storage

Raphael L. Streng, Samuel Reiser, Sabrina Wager, Nykola Pommer, and Aliaksandr S. Bandarenka*

Supporting Information for
A Fast and Highly Stable Aqueous Calcium-Ion Battery for Sustainable Energy Storage

Raphael L. Streng[1], Samuel Reiser[1], Sabrina Wager[1], Nykola Pommer[1],

Aliaksandr S. Bandarenka[1,2,*]

[1]*Physics of Energy Conversion and Storage, Physik-Department, Technische Universität München, James-Franck-Str. 1, 85748 Garching, Germany*

[2]*Catalysis Research Center TUM, Ernst-Otto-Fischer-Straße 1, 85748 Garching, Germany*

[*]Corresponding author.
E-mail: bandarenka@ph.tum.de (A.S. Bandarenka)

1. Structural Characterization of Poly(Naphthalene Four Formyl Ethylenediamine) (PNFE)

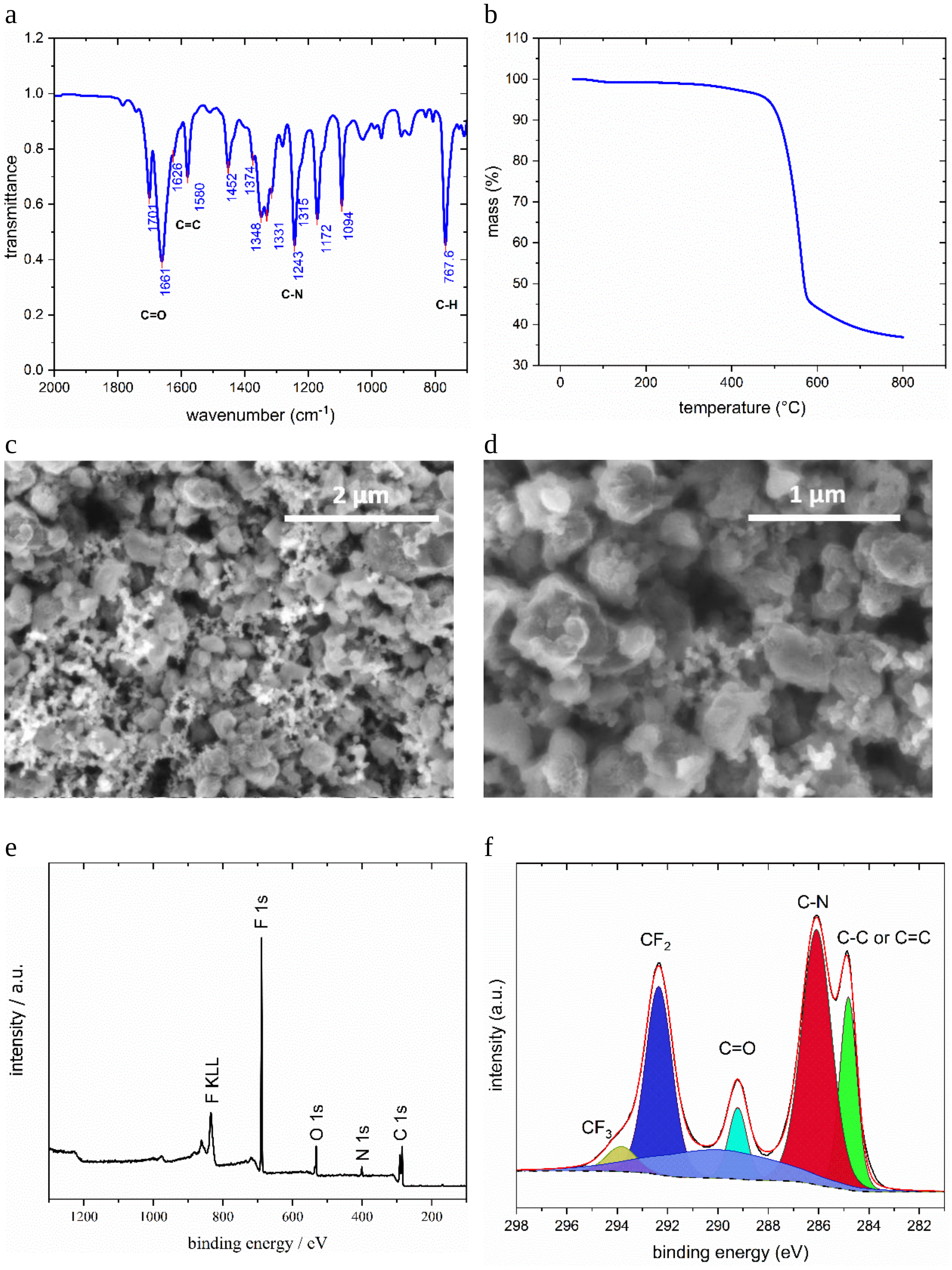

Figure S1. *Structural characterization of PNFE. (a) Fourier-transform infrared (FTIR) spectrum. (b) Thermogravimetric analysis curve. (c), (d) Scanning electron microscope pictures. (e) XPS survey spectrum of the PNFE electrode. (f) Fitted XPS C 1s spectrum of the PNFE electrode. (Replotted from our previous work [1])*

2. Structural Characterization of copper hexacyanoferrate (CuHCF)

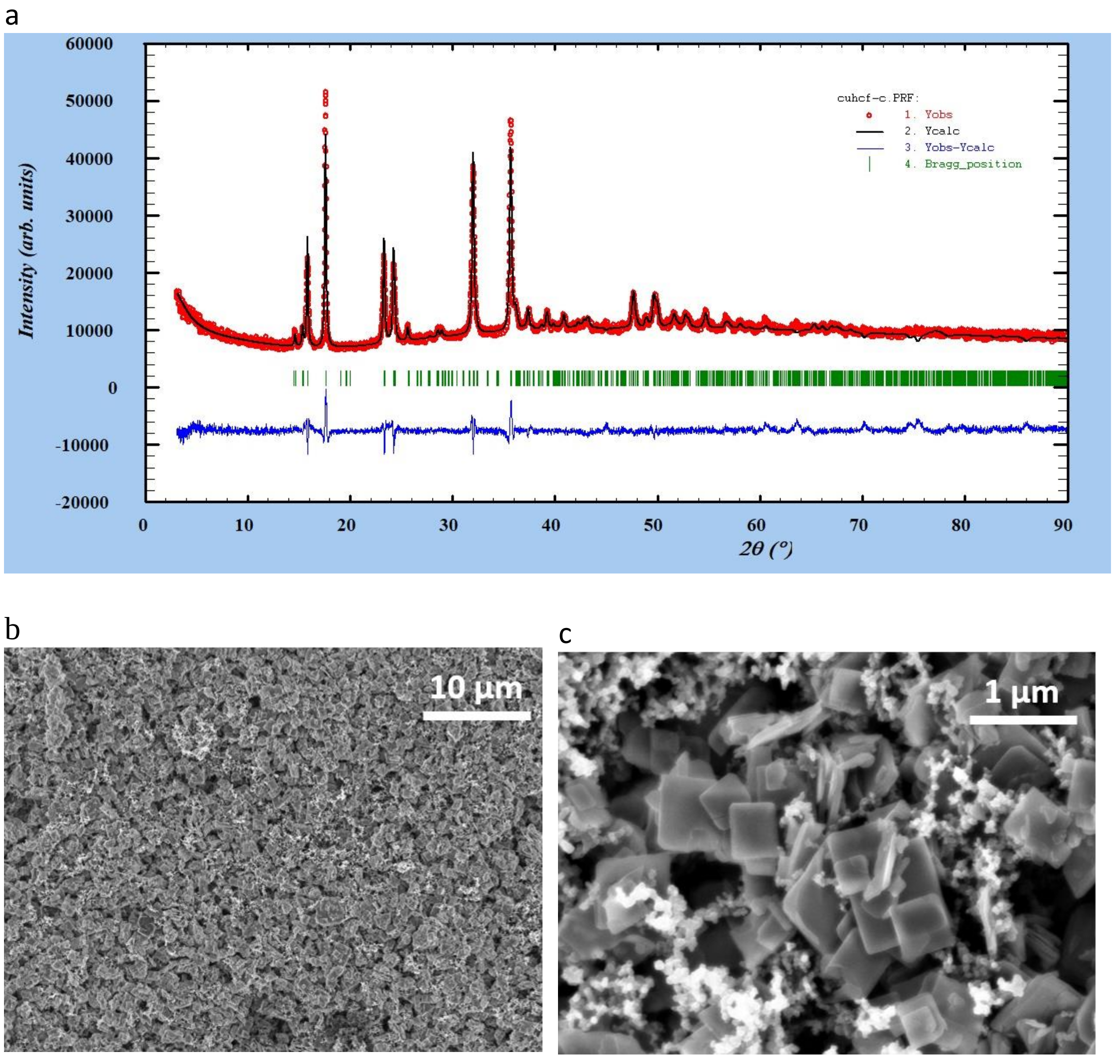

Figure S2. *Structural characterization of CuHCF. (a) Fitted x-ray diffraction spectrum corresponding to a monoclinic structure. (c), (d) Scanning electron microscope pictures.*

3. Structural stability of the Electrode Materials Upon Ball Milling

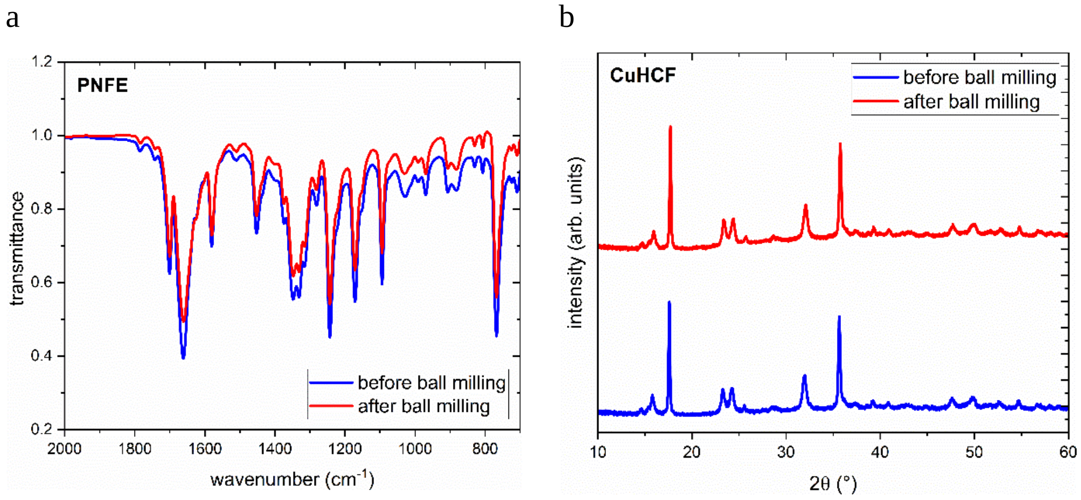

Figure S3. *Structural stability of the electrode materials upon ball milling. (a) FTIR spectrum of PNFE before and after ball milling. (b) XRD spectrum of CuHCF before and after ball milling.*

3. Comparison of the aqueous Ca-ion battery to similar systems

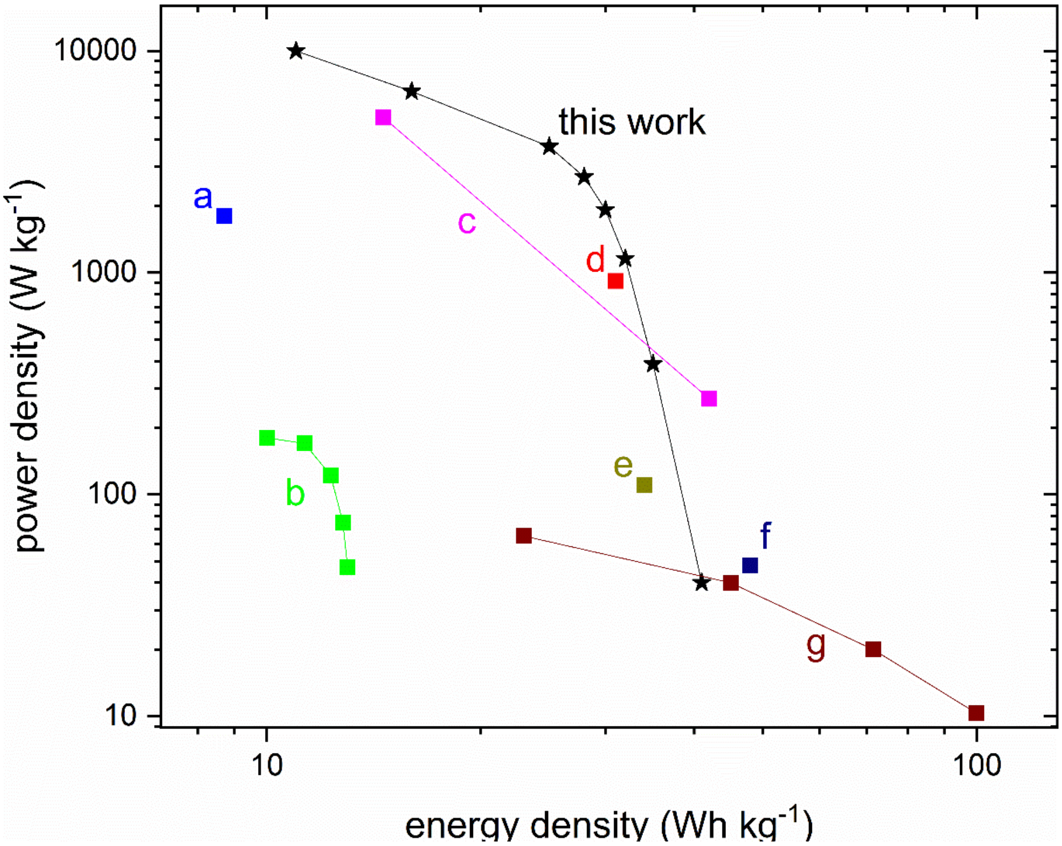

Figure S4. *Comparison of the electrochemical performance of the full cell in this work with other aqueous Ca-ion batteries reported by (a) Li et al. (2023) [2], (b) Adil et al. (2020) [3], (c) Wang et al. (2024) [4], (d) Zhang et al. (2023) [5], (e) Zhou et al. (2022) [6], (f) Gheytani et al. (2017) [7], and (g) Tang et al. (2021) [8]. The energy and power densities are normalized to the anode and cathode active material mass. If not stated directly, they are calculated based on the values reported in the respective references.*

References

[1] R. L. Streng, S. Vagin, Y. Guo et al., “Identifying the charge storage mechanism in polyimide anodes for Na-ion aqueous batteries by impedance spectroscopy,” *Energy Advances*, vol. 3, no. 4, pp. 874–882, 2024.

[2] L. Li, G. Zhang, X. Deng et al., “A covalent organic framework for high-rate aqueous calcium-ion batteries,” *Journal of Materials Chemistry A*, vol. 10, no. 39, pp. 20827–20836, 2022.

[3] M. Adil, A. Sarkar, A. Roy et al., “Practical Aqueous Calcium-Ion Battery Full-Cells for Future Stationary Storage,” *ACS applied materials & interfaces*, vol. 12, no. 10, pp. 11489–11503, 2020.

[4] C. Wang, R. Li, Y. Zhu et al., “A Pyrazine‐Pyridinamine Covalent Organic Framework as a Low Potential Anode for Highly Durable Aqueous Calcium‐Ion Batteries,” *Advanced Energy Materials*, vol. 14, no. 1, 2024.

[5] S. Zhang, Y.-L. Zhu, S. Ren et al., “Covalent Organic Framework with Multiple Redox Active Sites for High-Performance Aqueous Calcium Ion Batteries,” *Journal of the American Chemical Society*, vol. 145, no. 31, pp. 17309–17320, 2023.

[6] R. Zhou, Z. Hou, Q. Liu et al., “Unlocking the Reversible Selenium Electrode for Non‐Aqueous and Aqueous Calcium‐Ion Batteries,” *Advanced Functional Materials*, vol. 32, no. 26, 2022.

[7] S. Gheytani, Y. Liang, F. Wu et al., “An Aqueous Ca-Ion Battery,” *Advanced science (Weinheim, Baden-Wurttemberg, Germany)*, vol. 4, no. 12, p. 1700465, 2017.

[8] X. Tang, D. Zhou, B. Zhang et al., “A universal strategy towards high-energy aqueous multivalent-ion batteries,” *Nature Communications*, vol. 12, no. 1, p. 2857, 2021.
